# Bilateral tuberculous psoas abscess

**DOI:** 10.1099/acmi.0.000135

**Published:** 2020-05-18

**Authors:** Mouhcine Miloudi, Lamiae Arsalane, Anas Kharrab, Youssef El Kamouni, Said Zouhair

**Affiliations:** ^1^​ Department of Microbiology, Avicenne Military Hospital, Marrakech, Morocco; ^2^​ Departement of Rhumatology, Avicenne Military Hospital, Marrakech, Morocco

**Keywords:** psoas abscess, spondylodiscitis, tuberculosis

## Abstract

The unilateral psoas abscess is a rare disease that is often caused by common germs, including *Staphylococcus aureus *. Tuberculous origin and bilateral involvement are even rarer, especially in developed countries. It may be primary or secondary to a neighbourhood focus. We report a case of bilateral tuberculous abscess of psoas in an immunocompetent patient secondary to spondylodiscitis.

## Introduction

Psoas muscle abscess is a relatively rare condition that is difficult to diagnose because of the polymorphism and non-specificity of clinical signs. It may be primary or secondary to intra-retroperitoneal or osteo-articular infection. The most frequently found germs are *Staphylococcus aureus* in primary abscesses and *Escherichia coli* [1] in secondary abscesses. Tuberculous origin is rare, especially in developed countries, and it is often unilateral; bilateral involvement is even rarer and mainly occurs in immunocompromised subjects [2]. We report a case of bilateral tuberculous abscess of psoas secondary to spondylodiscitis in an immunocompetent subject.

## CASE PRESENTATION

**Fig. 1.**
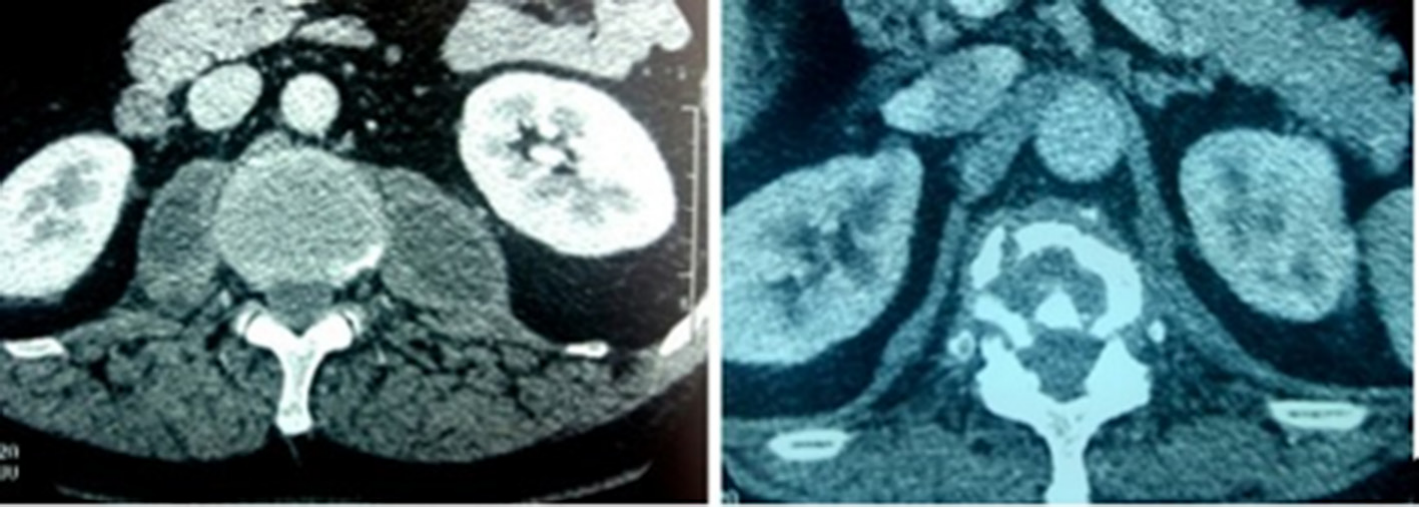
Abscess of both psoas with spondylodiscitis on a cross -section (CT).

The patient was 46 years old and had no pathological history, but 2 months before the consultation had inflammatory-type lumbar pain that developed in the context of deterioration of general condition. The interrogation revealed a possibility of tuberculous contagion 6 months before, and the clinical examination revealed lumbar pain on palpation and spinal stiffness without neurological attack. Abdominal–pelvic computed tomography (CT) showed spondylodiscitis at the level of the 10th, 11th and 12th dorsal vertebrae and the first lumbar vertebra with bilateral abscess collections at the expense of both psoas muscles (Fig. 1). The chest X-ray was normal. The biological assessment showed a C-reactive protein (CRP) level of 58 mg l^−1^ and a sedimentation rate of 60 mm/first hour, while the HIV serology was negative and the interferon gamma release assay (IGRA) test (Quantiféron) was positive.

**Fig. 2.**
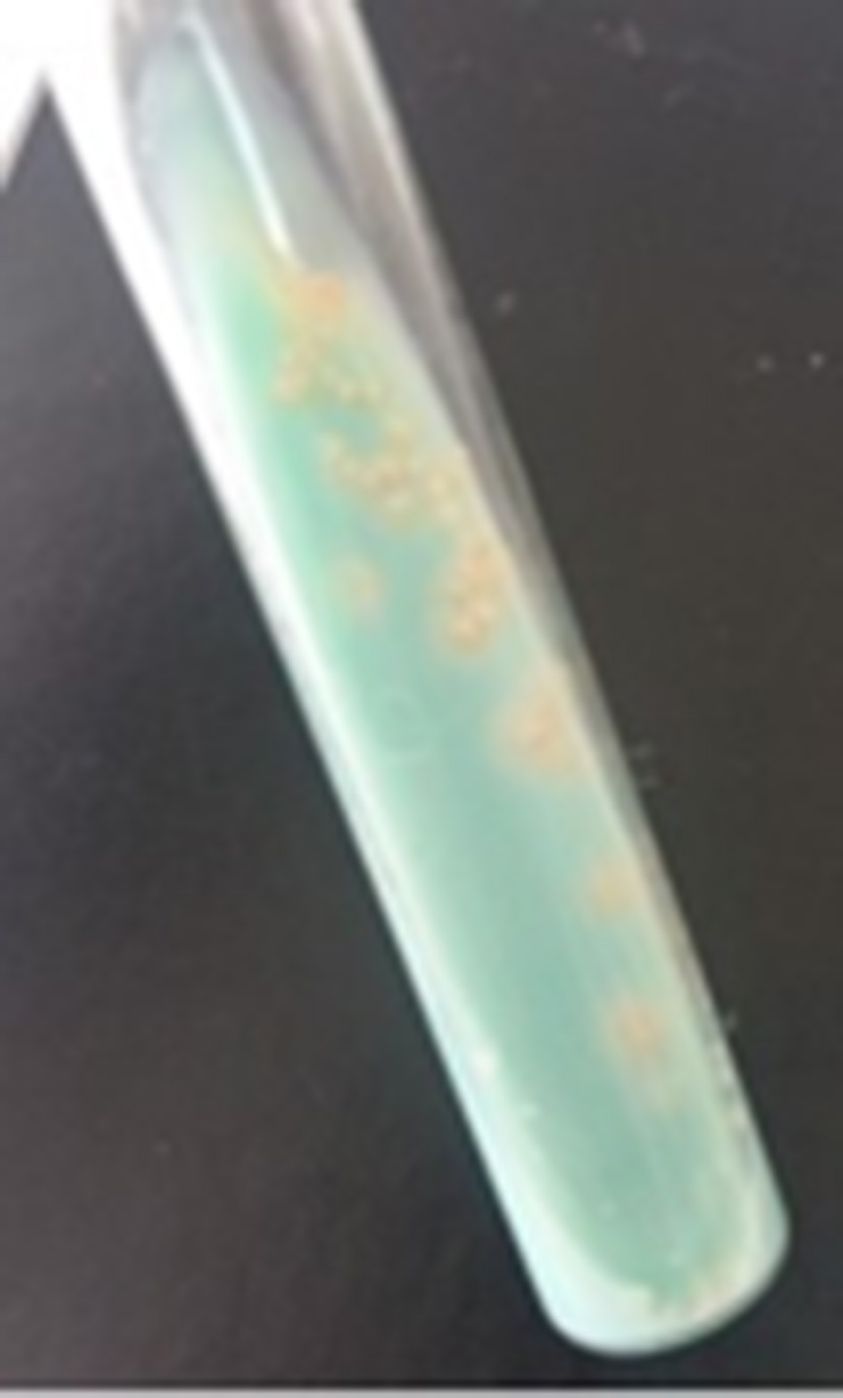
Beige verrucous colonies of *M. tuberculosis* on Lowenstein–Jensen medium.

The patient benefited from scanned/guided drainage and the pus brought back was subjected to bacteriological study (culture on blood agar and chocolate agar incubated at 37 °C in an aerobic and anaerobic atmosphere). This culture remained sterile after 4 days of incubation. For direct examination after Ziehl–Armand staining in search of acid-fast bacilli, the result was negative. The real-time PCR-based *Mycobacterium tuberculosis* test on the GeneXpert MTB/RIF system (which also makes it possible to detect rpoB gene mutations associated with rifampicin resistance), the system did not detect any mutations, so the strain was sensitive to rifampicin. A culture grown on solid Lowenstein–Jensen medium developed a month later (Fig. 2), while a culture grown in liquid medium (VersaTREK broth Myco Broth) and incubated in the VersaTREK automaton, whose technology is based on the detection of oxygen levels consumed by the mycobacteria, became positive after 20 days. When the susceptibility of the isolated strain to rifampicin, isoniazid and ethambutol was studied using the liquid dilution method on the versaTREK automaton, the strain was found to be sensitive to these antibiotics.

Following these results, the patient was put on rifampin, isoniazid, ethambutol and pyrazinamide for a duration of 2 months and rifampin and isoniazid for 10 months. The evolution was favourable, with weight recovery, loss of pain and decrease in CRP and sedimentation rate.

## DISCUSSION

Psoas abscess is a rare entity; the annual global incidence was estimated at 12 cases in 1992 [3], although the current incidence is unknown. This infection can be primitive by haematogenous dissemination from a distant focus, or secondary to retro, intraperitoneal (digestive, renal) or osteoarticular infection. Spondylodiscitis may be responsible for psoas abscess in 10 % of cases [4]. The offending organisms are often *S. aureus* and *Enterobacteriaceae* (* Escherichia coli *, *Klebsiella pneumoniae *), and tuberculous origin remains exceptional in developed countries but is still relevant in endemic countries such as Morocco, where tuberculosis is a major public health problem, with an incidence of 36 000 cases in 2017.

Osteoarticular tuberculosis represents 3–5 % of cases of tuberculosis and 15 % of cases of extrapulmonary tuberculosis. It affects all segments, but lumbar spine involvement remains most common [5]. Psoas involvement in spondylodiscitis is seen in 5 % of cases [6]; it is often unilateral and bilateral involvement is rare and mainly occurs in immunocompromised subjects. The clinical picture is insidious and nonspecific, which leads to delayed diagnosis and treatment; the fever triad, lumbar pain and hip stiffness are present in <50 % cases [7].

CT is the key examination for positive diagnosis; the biological signs are not very contributive and show an inflammatory syndrome and confirmation is achieved by bacteriological study of drainage product without forgetting to search for *M. tuberculosis *, which must be systematic in our context where tuberculosis is still endemic.

The treatment consists of percutaneous or surgical drainage combined with antituberculous treatment for a minimum of 12 months, combining rifampicin, isoniazid, ethambutol and pyrazinamide for 2 months, followed by 10 months of rifampicin and isoniazid.
